# Soothing liver-qi stagnation method for cancer-related depression: A protocol for systematic review and meta-analysis

**DOI:** 10.1097/MD.0000000000022797

**Published:** 2020-10-23

**Authors:** Jianfeng Zhang, Yunxia Liu, Yefeng Xu

**Affiliations:** a Zhejiang Chinese Medical University; b Department of Oncology, The Third People's Hospital of Hangzhou, Hangzhou, Zhejiang, China.

**Keywords:** cancer, depression, meta-analysis, protocol, soothing liver-qi stagnation method, systematic review, traditional Chinese medicine

## Abstract

**Background:**

Cancer-related depression (CRD) is the most common mood disorder in patients with malignant tumors, negatively influencing the patient's daily life. Traditional Chinese medicine, as an alternative CRD therapy, has shown good treatment performance in recently years. Soothing liver-qi stagnation, as a classic therapy for depression, is based on traditional Chinese medicine theory. However, there is no evidence-based medical confirmation for the soothing liver-qi stagnation method for CRD treatment.

**Methods:**

We will systematically search relevant articles from their inception to July 1, 2019 in the following electronic databases: the Cochrane Library, PubMed, EMBASE, Chinese National Knowledge Infrastructure Database, Chinese Biomedical Literature Database, Chinese Science and Technique Journals Database, and the Wan-fang Database. The primary outcome is the total scores of the Hamilton rating scale for depression and, the efficacy rate of reducing Hamilton rating scale for depression scores. The secondary outcomes are adverse reactions and quality of life as assessed by standard instruments. Two researchers will independently perform study selection, data extraction, and quality assessment. If there is any disagreement, it will be settled through third-party negotiations. We will assess the risk of bias and data synthesis using Review Manager (the Cochrane Collaboration) software, Version 5.3.0.

**Results:**

This work will evaluate the clinical effectiveness and safety of the soothing liver-qi stagnation method for CRD.

**Conclusion:**

This study may provide evidence-based medical corroboration for clinical application of the soothing liver-qi stagnation method for CRD treatment.

**PROSPERO registration number:**

CRD42019145678.

## 1 Introduction

Cancer is one of the leading factors threatening global human health. Due to a cancer diagnosis, patients experience long-term mood disorders. Of the various influences the disease has on patients’ psychological state, depression is one of the leading causes of psychological distress.^[1]^ Cancer-related depression (CRD) is a common comorbidity in patients with cancer that can occur in patients with any type of cancer, and its incidence is approximately 58%,^[2]^ with prevalence rates up to 4-times higher than in the general population.^[3]^ Meanwhile, according to a recent study,^[4]^ younger and female patients seem to be more affected by depression. However, oncologists pay less attention to patients’ latent emotional distress than to treatment plans and physical symptoms.^[5]^ CRD has become an inevitable health problem in modern society. Studies have found that depression in cancer patients increase hospitalization time and mortality, lowers quality of life, decreases compliance, and results in worse prognosis.^[6]^

Both pharmacotherapy and psychological treatment are effective for treating depression in cancer patients. According to current evidence, it is difficult to determine first-line recommendations for CRD treatment.^[7]^ Selective serotonin reuptake inhibitors, serotonin and noradrenaline reuptake inhibitors, agomelatine, bupropion, mirtazapine, and vortioxetine are all antidepressant medications, used extensively to alleviate moderate/severe depression.^[8]^ However, these drugs produce a variety of undesirable adverse effects such as headaches, fatigue, nausea, and dry mouth.^[8]^ Although new drugs targeting the neuroendocrine system, cytokines, or pro-inflammatory signaling have played a role in solving the aberrant pathways linking depression and cancer,^[9]^ the occurrence of severe gastrointestinal or cardiovascular events has haltered clinical implantation of these new drugs.

Over the past 2000 years, traditional Chinese medicine (TCM) has gained a deep understanding of depression, while herbal medicines have been widely applied for the treatment of depression. In the light of TCM theories, the activity of the liver-qi is thought to be related to the pathogenesis of emotional disease. Based on this assumption, making people's liver-qi smooth is key to treating patients with depression, or anxiety. For example, the formula of Chai Hu Shu Gan San, described in the Chinese ancient book Jing Yue Quan Shu, by Jingyue Zhang of the Ming dynasty, is being increasingly used for the treatment of depression.

Recent meta-analyses,^[10,11]^ showed that Chinese herbal medicine is a potential alternative therapy for depression. However, these meta-analyses just evaluated its effects from the aspects of herb medicines application and composition. To date, no systematic review has reported the differences in TCM therapeutic principles among studies in relieving the symptoms of cancer patients with depression. Therefore, we found it necessary and meaningful to perform a study to provide evidence-based medical proof for the clinical application of the soothing liver-qi stagnation method for CRD.

## 2 Methods

### 2.1 Protocol and registration

The study protocol was registered with the International Prospective Register of Systematic Review (PROSPERO), with registration number was CRD42019145678. This protocol was prepared in accordance with the Preferred Reporting Items for Systematic Reviews and Meta-analyses guidelines.^[12]^

### 2.2 Eligibility criteria

Randomized controlled trials to assess the effects of Chinese herbal medicine for soothing liver-qi stagnation in CRD will be included.

#### 2.2.1 Types of participants

The participants will be included to meet the following criteria: age ≥18 years; patients diagnosed with cancer regardless of cancer type, whether they received surgery, radiation, or chemotherapy, gender, race, or location; depression diagnosed according to the classification and diagnosis of mental disorders-3,^[13]^ Diagnostic and Statistical Manual-IV,^[14]^ and International Classification of Disease-10 (World Health Organization).^[15]^

#### 2.2.2 Type of interventions

All types of herbal medicines able to sooth liver-qi stagnation are included. There are no limitations on number, delivery method (eg, oral, intravenous, or external use), dosage, or treatment duration.

#### 2.2.3 Type of outcome measures

Primary outcomes: The total scores of the Hamilton rating scale for depression,^[16]^ and the efficacy rate of reducing those. Secondary outcomes: adverse reactions and quality of life assessed using standard quality of life instruments.

### 2.3 Information sources and search strategy

We will search the following electronic databases: The Cochrane Library, PubMed, EMBASE, Chinese National Knowledge Infrastructure Database, Chinese Biomedical Literature Database, Chinese Science and Technique Journals Database, and the Wan-fang Database. The search terms are the following: “depression,” “depressive disorder,” “depressed,” “affective disorder,” “mood disorder,” “mental health,” “melancholia,” “cancer,” “tumor,” “sarcoma,” “neoplastic,” “leukemia,” “lymphoma,” “malignant,” “soothing liver-qi stagnation,” “soothing the liver,” “dispersing stagnated liver qi,” “Radix Bupleuri,” “Rhizoma Cyperi,” “Rhizome of Chuanxiong,” “Cortex Albiziae,” “blind,” “placebo,” and “random.” We will systematically search relevant articles from their inception to July 1, 2019 in 7 electronic databases. No language or publication limitations. We have listed the detailed search strategies using the PubMed database as an example in Table 1. We will also search manually for relevant literature.

**Table 1:**
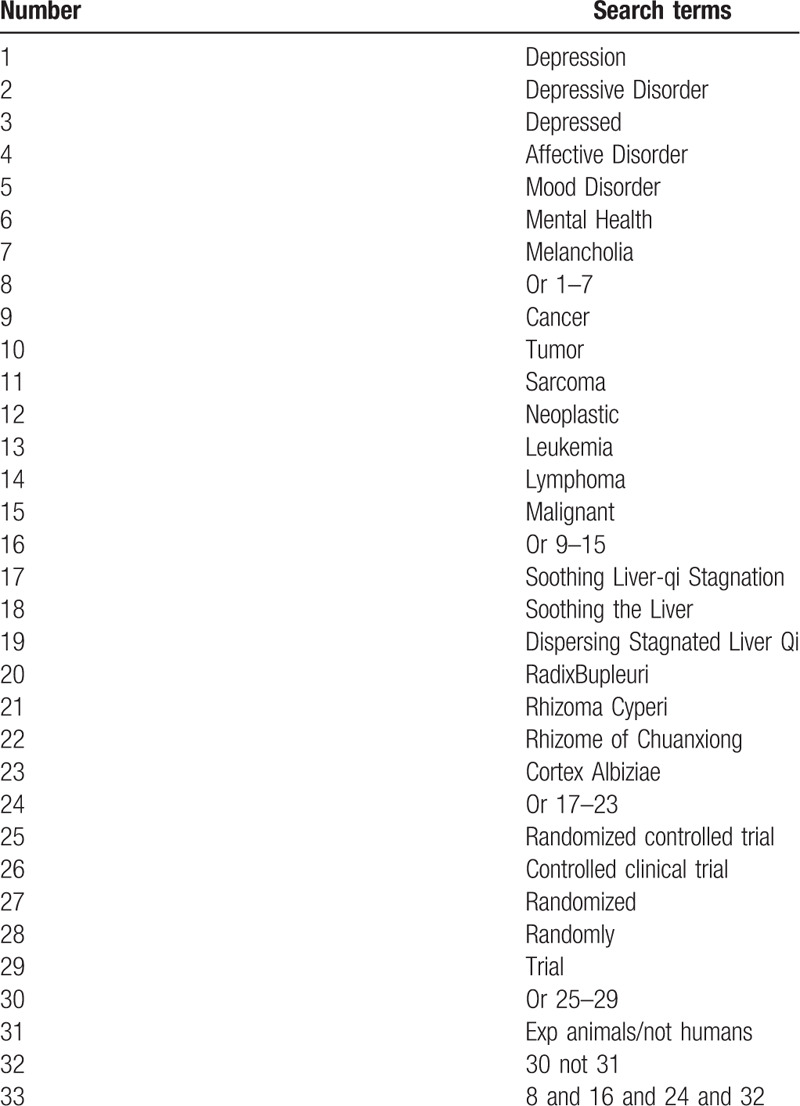
Search strategy for the PubMed database.

### 2.4 Study selection and data collection

**Figure 1:**
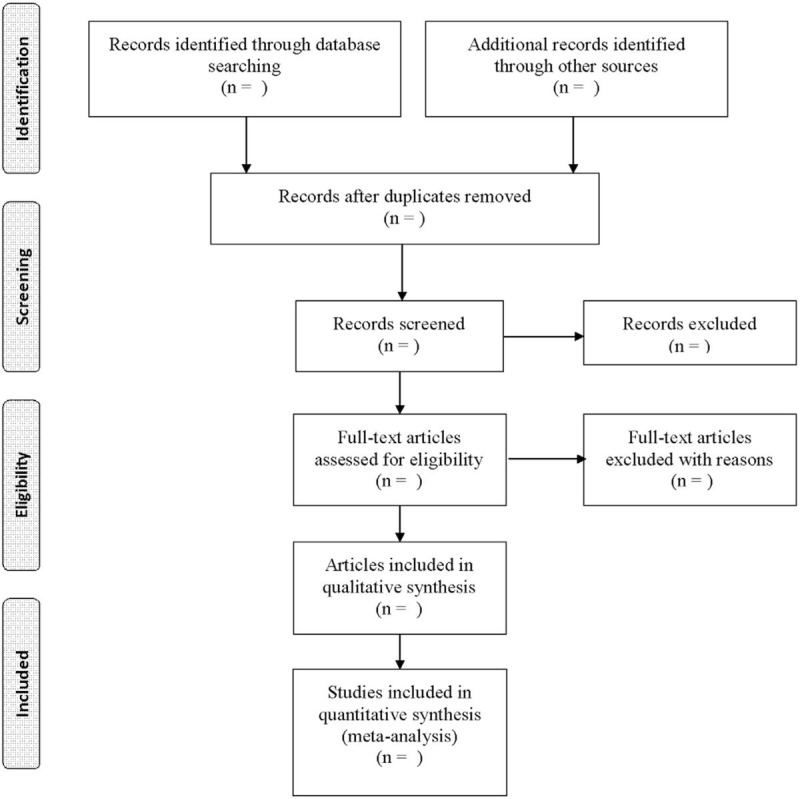
Process of study selection.

First, 2 researchers will search articles independently according to the search strategy. Then they will scan the titles and summary of retrieved articles eliminate those not meeting the requirements. In addition, to ensure that all articles fit the inclusion criteria, the whole pre-screened articles will be read again. The reasons for rejection are recorded separately at each step. Figure 1 shows the process flow chart. If there are any disagreements, they should first negotiate with each other. If necessary, a third investigator will address the divergence. Finally, the 2 researchers will independently extract data such as general trial characteristics (first author, year of publication), baseline data (age, gender, and sample size), treatment details (dose, treatment course), and outcome (outcome measures, adverse events).

### 2.5 Assessment of risk of bias

Two authors will independently assess the risk of bias in trials according to the Cochrane Handbook for Systematic Reviews of interventions. The following items will be assessed: random sequence generation, allocation concealment, blinding of participants and personnel, blinding of outcome assessment, incomplete outcome data, selective reporting, and other sources of bias. Discrepancies in the interpretation will be resolved by consensus or with the involvement of a third party.

### 2.6 Data analysis

We will review the data with Review Manager (Cochrane Collaboration) software, Version

5.3.0. For dichotomous data, we will use the risk ratio with 95% confidence intervals (CI); and mean difference with 95% CI for continuous data. If different measurement scales are used, standardized mean difference analysis with 95% CI will be performed. The Chi-square test and the Higgins *I*^*2*^ test will be used to assess heterogeneity. A fixed-effects model will be used if no statistical heterogeneity is found among studies (*P* > .10, *I*^*2*^ < 50%), and a random-effects model employed if significant heterogeneity is found (*P* ≤ .10, *I*^*2*^ ≥ 50%). If the heterogeneity is significantly high, we will analyze the source of heterogeneity, and a subgroup or descriptive analysis may be taken.

#### 2.6.1 Subgroup analysis

Subgroup analysis may follow these aspects: type of control group intervention (eg, placebo, no treatment or conventional western medicine), forms of Chinese herb medicine (eg, decoctions, tables, capsules, pills, or powders), and mode of herbal delivery (eg, oral, intravenous, or external use).

#### 2.6.2 Sensitivity analysis

A sensitivity analysis will be performed to assess the reliability of the conclusion by changing the effect model, statistical methods, and eliminating high-risk bias studies.

#### 2.6.3 Publication bias

Once the subgroup includes ≥10 randomized controlled trials, funnel plots will be used to assess reporting bias. We will evaluate the existence of publication bias by the symmetry of the funnel plot and try to interpret the probable cause.

### 2.7 Confidence in cumulative Evidence

The Grading of Recommendations Assessment, Development, and Evaluation will be used to assess the quality of evidence for the various outcomes.

### 2.8 Ethics and dissemination

Because our data are based on published articles, no ethical approval is required in this systematic review. In addition, the findings of this systematic review will be published through peer-review publications.

## 3 Discussion

In recent years, the mental health of cancer patients has gained more attention.^[17]^ Depression is one of the most common psychological disorders in cancer patients, and both pharmacotherapy and psychological treatment have positive effects, although treatment discontinuation and failure are common.^[18]^ However, for cancer patients with varying degrees of depression, there is no sufficient evidence-based medical evidence for reference on treatment strategies based on pharmacotherapy and psychological treatment.^[19,20]^ Chinese herbal medicine, a low-cost, and convenient form of medicine, has gradually become the preferred alternative therapy for treating depression in cancer patients.^[10]^

As mentioned earlier, previous meta-analyses^[10,11]^ have verified the effectiveness of Chinese herbal medicine in treating cancer patients with depression, but only from the perspective of drug composition or prescription, not taking into consideration the differences in TCM therapies among studies. A tailored treatment based on each particular TCM syndrome is the basic feature of TCM theory and, the basis for all TCM doctors to carry out clinical diagnosis and treatment.^[21,22]^ The meaningful procedure of TCM diagnosis and treatment is to identify the corresponding treatment principles according to individual symptoms. Therefore, it is necessary to develop strict retrieval strategies to conduct high-quality research on the treatment of CRD with soothing liver-qi stagnation therapy. The purpose of this systematic review is to summarize and evaluate the evidence on a large number of Chinese herbal medicine prescriptions with the function of soothing liver-qi stagnation for CRD treatment in the hope that the results may provide an informed alternative for future clinical applications.

## Author contributions

**Conceptualization:** Jianfeng Zhang, Yunxia Liu.

**Data curation:** Jianfeng Zhang, Yefeng Xu.

**Funding acquisition:** Yunxia Liu, Yefeng Xu.

**Methodology:** Jianfeng Zhang, Yefeng Xu.

**Project administration:** Yunxia Liu.

**Supervision:** Yunxia Liu, Yefeng Xu.

**Writing – original draft:** Jianfeng Zhang.
